# MRI Screening for Optic Gliomas in Neurofibromatosis Type 1

**DOI:** 10.15844/pedneurbriefs-29-9-7

**Published:** 2015-09

**Authors:** J. Gordon Millichap

**Affiliations:** 1 Division of Neurology, Ann & Robert H. Lurie Children's Hospital of Chicago, Chicago, IL; 2 Departments of Pediatrics and Neurology, Northwestern University Feinberg School of Medicine, Chicago, IL

**Keywords:** Neurofibromatosis Type 1, Optic Pathway Gliomas, Brain/Orbit MRI

## Abstract

Investigators from Cincinnati Children's Hospital, OH, analysed retrospectively the utility of screening brain/orbital MRIs in 826 children with NF1 (ages 1-9 years; 402 female, 424 male) seen over a 20-year period between 1990 and 2010.

Investigators from Cincinnati Children's Hospital, OH, analysed retrospectively the utility of screening brain/orbital MRIs in 826 children with NF1 (ages 1-9 years; 402 female, 424 male) seen over a 20-year period between 1990 and 2010. Baseline MRIs of brain and orbits with and without contrast were obtained at 15 months of age or at the time NF1 diagnosis was made. Children identified with OPG were followed with repeat MRI every 3-6 months until OPG was stable. Other patients had annual eye exams and were seen by NF team. Optic pathway gliomas (OPGs) were identified in 18%, with a median age at detection of 3 years. Of 149 with OPGs, 96 (64.4%) were prechiasmatic, 42 (28.2%) chiasmatic, and 11 (7.4%) postchiasmatic; 22 (15%) had radiological or clinical progression requiring therapy. Chiasmatic and postchiasmatic tumors required therapy more frequently than prechiasmatic OPGs (P<0001). Patients with visual deficits at diagnosis (12/22) were more likely to have visual decline despite therapy when compared with patients treated based on radiologic progression (P<.012). Hypopituitarism (6/22) and precocious puberty (5/22) were common comorbidities of patients with chiasmatic and postchiasmatic OPGs and were not a feature of prechiasmatic tumors. Time to therapy after MRI diagnosis ranged from 0.2 and 5 years. Early identification of OPG by screening MRI before the development of vision loss may lead to improved visual outcomes. Children with negative brain and orbital MRI screening at age 15 months or later did not develop symptomatic OPGs. [1]

COMMENTARY. Based on the results of this study the authors advocate the routine MRI screening of brain and orbits of children with NF1 [1]. This opinion is in agreement with a 2004 study and report of benefits of MRI screening of 84 children with NF1 [2], and is contrary to an authority who, in 2004, recommended screening only with ophthalmological examinations in young asymptomatic children [3]. In 1997, the OPG Task Force concluded that early detection of tumors would not reduce the rate of loss of vision, and there was no evidence to support MRI screening with MRI [4]. Perhaps, the current data will lead to a further meeting of a Task Force and a change in guide-lines for the management of OPGs, with inclusion of brain/orbit MRIs in children with neurofibromatosis type l.
